# Overexpression of matrix metalloproteinases and their inhibitors in mononuclear inflammatory cells in breast cancer correlates with metastasis-relapse

**DOI:** 10.1038/sj.bjc.6603963

**Published:** 2007-09-11

**Authors:** L O González, I Pidal, S Junquera, M D Corte, J Vázquez, J C Rodríguez, M L Lamelas, A M Merino, J L García-Muñiz, F J Vizoso

**Affiliations:** 1 Unidad de Investigación, Hospital de Jove, Gijón, Spain; 2 Instituto Universitario de Oncología del Principado de Asturias, Asturias, Oviedo, Spain; 3 Servicio de Anatomía Patológica, Hospital de Jove, Gijón, Spain; 4 Servicio de Cirugía General, Hospital de Jove, Gijón, Spain; 5 Servicio de Ginecología, Hospital de Jove, Gijón, Spain; 6 Servicio de Anatomía Patológica, Hospital de Cabueñes, Gijón, Spain; 7 Servicio de Cirugía General, Hospital Universitario Central de Asturias, Asturias, Oviedo, Spain

**Keywords:** breast cancer, prognosis, MMP, TIMP, tumoural invasion, metastasis

## Abstract

An immunohistochemical study was performed using tissue microarrays and specific antibodies against matrix metalloproteinase (MMP)-1, -2, -7, -9, -11, -13 and –14, tissular inhibitors of metalloproteinase (TIMP)-1, -2 and -3. More than 2600 determinations on cancer specimens from 131 patients with primary ductal invasive tumours of the breast were performed. To identify specific groups of tumours with distinct expression profiles the data were analysed by unsupervised hierarchical cluster analysis by each cellular type. We did not find well-defined cluster of cases for tumour cells or fibroblastic cells. However, for mononuclear inflammatory cells the dendogram shows a first-order division of the tumours into two distinct MMP/TIMP molecular profiles, designated group 1 (*n*=89) and group 2 (*n*=42). Matrix metalloproteinase-7, -9, -11, -13 and -14, and TIMP-1 and -2, were identified as showing significant high expression in group 2 compared with group 1. Multivariate analysis demonstrated that clustering for mononuclear inflammatory cells was the most potent independent factor associated with distant relapse-free survival (group 2: 5.6 (3.5–9.6), *P*<0.001). We identify a phenotype of mononuclear inflammatory cells infiltrating tumours, which is associated with the development of distant metastasis. Therefore, this finding suggests that these host inflammatory cells could be a possible target for inhibition of metastasis.

Degradation of the stromal connective tissue and basement membrane components are key elements in tumour invasion and metastasis. Some components of the extracellular matrix, particularly the interstitial collagens, are very resistant to proteolytic attacks, being degraded only by matrix metalloproteinases (MMPs) (Nelson *et al*, 2000). Matrix metalloproteinases are also able to impact on tumour cell behaviour *in vivo* as a consequence of their ability to stimulate the tumoural growth, antiapoptotic factors, motility cellular or angiogenesis (Manes *et al*, 1999; Rifkin *et al*, 1999; Fingleton *et al*, 2001; Noe *et al*, 2001; Sternlicht and Werb, 2001; Egeblad and Werb, 2002; Turk *et al*, 2004).

Several MMPs, in particular the gelatinases MMP-2 (Jones *et al*, 1999; Duffy *et al*, 2000; Talvensaari-Mattila *et al*, 2001, 2003; Baker *et al*, 2002; Grieu *et al*, 2004; Li *et al*, 2004; Sivula *et al*, 2005) and MMP-9 (Chantrain *et al*, 2004; Li *et al*, 2004; Pellikainen *et al*, 2004), have been recently studied as prognostic factors in breast cancer, being associated with a poor outcome in various subsets of patients. Discordant data have also been published on the prognostic value of the above-mentioned MMPs (Jones *et al*, 1999; Hirvonen *et al*, 2003; Talvensaari-Mattila *et al*, 2003; Grieu *et al*, 2004; Sivula *et al*, 2005; Talvensaari-Mattila and Turpeenniemi-Hujanen, 2005). Nevertheless, it has been suggested that the coexpression of various MMPs and/or tissular inhibitors of metalloproteinases (TIMPs) might provide additional prognostic information in breast cancer. Accordingly, it has been reported that several other MMPs and TIMPs may be overexpressed and/or related to clinical outcome in breast cancer, such as MMP-11 (Duffy *et al*, 2000) MT1-MMP (MMP-14) (Jones *et al*, 1999; Mimori *et al*, 2001) MMP-13 (Nielsen *et al*, 2001), TIMP-1 (Ree *et al*, 1997; McCarthy *et al*, 1999; Nakopoulou *et al*, 2002a; Schrohl *et al*, 2003, 2004) or TIMP-2 (Visscher *et al*, 1994; Ree *et al*, 1997; Remacle *et al*, 2000). In addition, there are at least two elements adding more complexity to the role of MMPs and TIMPs in cancer. On the one hand, it is now assumed that TIMPs are multifactorial proteins also involved in the induction of proliferation and the inhibition of apoptosis (Baker *et al*, 1999; Jiang *et al*, 2002) TIMP-3 (Guedez *et al*, 2001) and TIMP-1. On the other hand, we should consider that the cellular type (tumoural cell/stromal cell) expressing these factors might be of biological importance in breast cancer (see Decock *et al*, 2005, for review). Thus, for example, it has been demonstrated that positive stromal MMP-9 expression is related to HER-2 overexpression in oestrogen receptor-positive tumours and predicts a shorter relapse-free and overall survival, whereas MMP-9 expression in carcinomatous cells predicts a longer relapse-free survival (Pellikainen *et al*, 2004).

All these data suggest the importance of the consideration of new techniques that would allow evaluation of the expression of several MMPs and TIMPs by each cellular type of the tumoural scenario, to contribute to the understanding of the molecular complexity of breast cancer and to a more precise prognostic evaluation. Immunohistochemistry on tissue microarrays (TMAs) provide an efficient platform to observe protein expression in a large number of cases with a limited amount of reagents and with a short analysis time (Gulmann *et al*, 2003). Recently, we reported the clinical value of this technology to evaluate the expression of MMP-1, -2, -7, -9, -11, -13 and –14, and TIMP-1, -2 and 3, in breast cancer (Vizoso *et al*, 2007). In this study, we validated this method against the determination of the expression of these parameters in the whole-tissue sections of the tumours, and we also found significant associations between the expression of several MMPs and the occurrence of distant metastasis. Following with our investigations, the objectives of the present work were (i) to investigate the relationship between these several MMPs and TIMPs expressions by the cellular type (tumoural/stromal cells) and the clinicopathological and biological characteristics of the tumours; and (ii) to attempt the identification of the different phenotypes of tumour or stromal cells associated with the development of distant metastases.

## MATERIALS AND METHODS

### Patients’ selection

This study comprised 131 women with a histologically confirmed diagnosis of early invasive breast cancer of ductal-type treated between 1990 and 2001, which were previously included in our preliminary study on the expression of MMPs and TIMPs in breast cancer (Vizoso *et al*, 2007). This study population include a significant number of patients with relapse in both node-negative and node-positive patient's subgroups (half of cases with distant metastasis during the follow-up period in each one of these subgroups) for securing the statistical power of the survival analysis. Data about treatment and following of the patients were described elsewhere (Vizoso *et al*, 2007). The study adhered to national regulations and was approved by our Institution's Ethics and Investigation Committee.

### Tissue microarrays and immunohistochemistry

Routinely fixed (overnight in 10% buffered formalin), paraffin-embedded tumour samples stored in our pathology laboratory files were used in this study. Details about TMAs technique were described elsewhere (Vizoso *et al*, 2007). From the 131 tumour samples available, four tissue array blocks were prepared, each containing 33 tumour samples, as well as internal controls including four normal breast tissue samples from two healthy women that underwent reductive mammary surgery. Immunohistochemistry was carried out on sections of 5 *μ*m of TMA fixed in 10% buffered formalin and embedded in paraffin using a TechMate TM50 autostainer (Dako, Glostrup, Denmark). Antibodies for MMPs and TIMPs were obtained from Neomarker (Lab Vision Corporation, Fremont, CA, USA). The dilution for each antibody was established based on negative and positive controls (1 out of 50 for MMP-2, -7 and -14, TIMP-2 and -3; 1 out of 100 for MMP-1, -9 and -13 and TIMP-1; and 1 out of 200 for MMP-11). The immunohistochemistry technique was also described for us in a recent report (Vizoso *et al*, 2007).

### Tissue microarray analysis

For each antibody preparation studied, the location of immunoreactivity in each cellular type was determined. In each case, immunoreactivities were classified into two categories in each cell type (tumour, fibroblastic and inflammatory mononuclear cells), depending upon the percentage of cells stained (negative: 0–10% positive cells; positive: >10% positive cells). We studied both cores that were carried out for each patient and averaged the results. If there was no tumour in a particular core, then the results of the other was given. Staining was studied by two pathologists (LOG and AMM) blinded to the clinical outcome of the patients.

### Western blot analysis

Western blotting of metalloproteinases (MMP-1, -11, -13 and -14) from human breast cancer cytosolic extracts or from human placenta (used as positive control) was performed as follows: samples were subjected to 12% SDS–PAGE and transferred onto a nitrocellulose membrane for 1 h at room temperature. Nitrocellulose membrane was blocked with 2% non-fat dry milk in TBS (50 mM Tris–HCl, 100 mM NaCl, pH 8.0) with 0.1% Tween 20 for 1 h at room temperature. Blots were then immunolabelled overnight at 4°C with a monoclonal anti-MMP-11 (1 : 200, Lab Vision Corporation). After three washes for 5 min each in TBST, membranes were incubated with goat anti-mouse IgG (1 : 2500) (or 1 : 5000, anti-rabbit IgG for MMP-1 and -14, see below) peroxidase-conjugated second antibody, using the ECL™ Western blotting analysis system (Amersham Biosciences, CE Healthcare, Buckinghamshire, UK), and visualised by placing the blot in contact with standard X-ray film according to the manufacturer's instructions. Membranes were stripped by incubation in 0.2 M glycine, pH 2.2, containing 0.1% SDS and 1% Tween 20 at room temperature for 1 h, and then re-probed with an anti-MMP-1 (1 : 200, Lab Vision Corporation), anti-MMP-13 (1 : 100, Calbiochem, Barcelona, Spain), and anti-MMP-14 antiserum (1 : 200, Lab Vision Corporation), or an anti-*β*-actin monoclonal antibody (1 : 2500; Sigma-Aldrich, St Louis, MO, USA) to confirm that equivalent amounts of total protein were added to each well.

### Data analysis and statistical methods

Differences in percentages were calculated with the *χ*^2^ test. For metastasis-free survival analysis we used the Cox's univariate method. Cox's regression model was used to examine interactions of different prognostic factors in a multivariate analysis. Expression profiles were analysed by unsupervised hierarchical clustering method that organises proteins in a tree structure, based on their similarity. Data were reformatted as follows: −3 designated negative staining, 3 positive staining, missing data were left blank. The score values were reformatted (positive–negative) choosing the median as cutoff value. We used the Cluster 3.0 programme (average linkage, Pearson's correlation). Results were displayed with Treeview (Eisen *et al*, 1998). The SPSS 11.5 programme was used for all calculations.

## RESULTS

**Figure 1:**
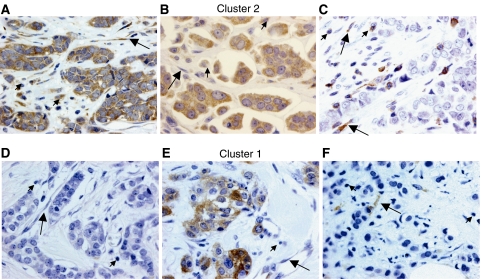
Left: examples of tissue with immunostaining for the more significant proteins in patients with tumours belong to poor prognostic group (cluster 2 group: **A**, **B** and **C**) and better prognostic group (cluster 1 group: **D**, **E** and **F**; magnification × 400). (**A**) Immunohistochemical staining of matrix metalloproteinase (MMP)-11 in inflammatory mononuclear cells, tumour cells and fibroblastic cells. (**B**) Immunohistochemical staining of MMP-9 in inflammatory mononuclear cells, tumour cells and fibroblastic cell negatives. (**C**) Immunohistochemical staining of tissular inhibitors of metalloproteinase (TIMP)-1 in inflammatory mononuclear cells and in fibroblastic cells, tumour cell negatives. (**D**) No immunohistochemical staining of MMP-9 in inflammatory mononuclear cells, tumour cells and fibroblastic cells. (**E**) Immunohistochemical staining of MMP-11 in tumour cells, inflammatory mononuclear cells and fibroblastic cell negatives. (**F**) Immunohistochemical staining of TIMP-1 in fibroblastic cells, inflammatory mononuclear cells and tumour cell negatives. In all cases, inflammatory mononuclear cells are indicated with small arrows, and fibroblastic cells with large arrows.

Figure 1 shows examples of tissue with immunostaining for proteins evaluated. The percentage of positive cells for each protein was always higher than 50% in positive case for each cellular type.

Matrix metalloproteinase-1 was immunohistochemically detected in 88.3% of breast carcinomas, MMP-2 in 42%, MMP-7 in 87.4%, MMP-9 in 74%, MMP-11 in 89.3%, MMP-13 in 73.3%, MMP-14 in 90%, TIMP-1 in 95%, TIMP-2 in 87% and TIMP-3 in 82.3%. However, there was also variability with regard to the cell type expressing each protein. For all proteins immunostaining was localised predominantly in tumour cells, but also in stromal cells (fibroblasts and/or mononuclear inflammatory cells) in significant percentages of cases for each studied protein.

**Figure 2:**
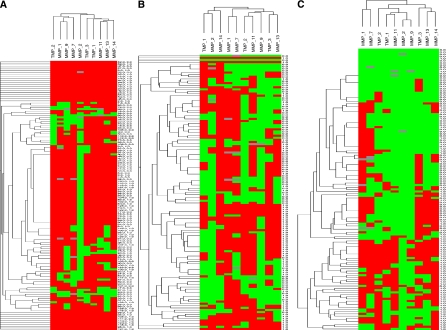
Hierarchical clustering analysis of global matrix metalloproteinases (MMPs)/tissular inhibitors of metalloproteinases (TIMPs) expressions in the different cell types of breast cancer as measured by immunohistochemistry on tissue microarray (TMA). Graphical representation of hierarchical clustering results in tumour cells (**A**), fibroblasts (**B**) and mononuclear inflammatory cells (**C**). Rows, tumoural samples; columns, MMPs/TIMPs. Protein expressions are depicted according to a color scale: red, positive staining; green, negative staining; grey, missing data. No major clusters of tumours are shown in tumour cells and fibroblasts. Two major clusters of tumours (1 and 2) are shown in mononuclear inflammatory cells.

**Figure 3:**
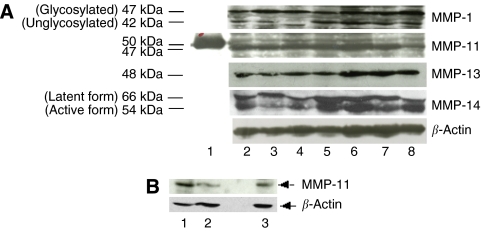
(**A**) Representative Western blots of immunoreactive matrix metalloproteinases (MMPs). (**A**) Eighty micrograms of cytosol extract from seven human breast carcinomas (lanes 2–8) were subject to 12% SDS–PAGE and transferred onto a nitrocellulose membrane and then immunolabelled with MMP-1, -11, -13, -14 and *β*-actin (used as loading control) antiserum. Lane 1, molecular weight marker (50 kDa). (**B**) Western blots of immunoreactive MMP-11 and *β*-actin (used as loading control) from two human breast carcinomas (lanes 1 and 2) and from human placenta (used as positive control, lane 3).

To identify specific groups of tumours with distinct MMP/TIMP immunohistochemical expression profiles the data were analysed by unsupervised hierarchical cluster analysis by each cellular type. The algorithm orders proteins on the horizontal axis and samples on the vertical axis based on similarity of their expression profiles. This did not produce a dendrogram with well-defined cluster of cases for tumour cells or fibroblastic cells (Figure 2A and B). However, for mononuclear inflammatory cells the dendogram shows a first-order division of the tumours into two distinct MMP/TIMP molecular profiles, designated group 1 (*n*=89) and group 2 (*n*=42) (Figure 2C). Matrix metalloproteinase-7, -9, -11, -13 and -14, and TIMP-1 and -2, were identified as showing significant high expression in group 2 compared with group 1 (Table 1). The presence of the proteins was confirmed by Western blot analysis of breast tumour cytosols samples (Figure 3). Western blots clearly showed immunoreactive bands corresponding to MMP-1, -11, -13 and -14 in seven samples from breast carcinomas (Figure 3A, upper panel). Two major immunoreactive bands were readily visible in samples from MMP-1 (glycosylated and unglycosylated form) and MMP-14 (latent and active form). As positive control, we also performed a Western blot for MMP-11 protein detection in placenta. As shown in Figure 3B, the anti-MMP-11 antibody recognised a band of approximately 47 kDa corresponding to the expected size in both, breast carcinomas (lanes 1 and 2), and placenta (lane 3).

**Figure 4:**
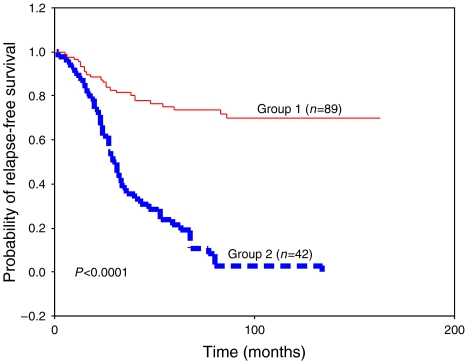
Kaplan–Meier survival curves as a function of the two major clusters of tumours (Groups 1 and 2) shown in mononuclear inflammatory cells.

These two groups showed significant differences with regard to PgR status and peritumoural inflammation. Group 1 showed a higher percentage of PgR-positive tumours, whereas group 2 showed a higher percentage of tumours with peritumoural inflammation. In addition, group 2 showed a significantly higher percentage of tumours positive for TIMP-1, MMP-9 and -11 in the tumour cells; as well as a significantly higher percentage of tumours positive for TIMP-2, TIMP-3, MMP-9, -11, -13 and -14, in fibroblastic cells (Table 2). However, our result did not show significant association between the cluster group of tumours with other clinicopathological characteristics, such as age, menopausal stage, tumour size, nodal status, histological grade, Nottingham Prognostic Index, oestrogen receptors, desmoplastic reaction, tumour advancing edge or vascular invasion (data not shown). On the other hand, during the study period, distant metastasis-relapse was confirmed in 41 of 42 (97.61%) patients of group 2 tumours and only in 24 of 89 (26.96%) patients of group 1 tumours. Kaplan–Meier analysis shows that the difference in relapse-free survival between these two groups is highly and statistically significant (*P*<0.0001; Figure 4). Multivariate analysis according to Cox model demonstrated that tumoural stage (II: (relative risk (RR) (confidence interval (CI)=1.6 (0.8–3.1); III: 3.5 (1.7–7.1); *P*<0.001) and ER status (positive: 0.5 (0.3–0.8), *P*<0.001) were significantly and independently associated with relapse-free survival. However, this same analysis also demonstrated that clustering for mononuclear inflammatory cells was the most potent independent factor associated with relapse-free survival (group 2: 5.6 (3.5–9.6), *P*<0.001).

## DISCUSSION

The results of the present study led us to several considerations, on the cellular type expressing each MMPs or TIMPs, the cellular origin of its production, the importance of stromal–tumour cell interactions, and the clinical value of these proteins not only in predicting outcome in breast cancer but also as a potential biological therapeutic target.

Breast cancer, as a solid tumour, consists of a variable mixture of neoplastic cells and non-neoplastic tumour stroma, comprising endothelial cells, pericytes, fibroblast cells and variable representation of inflammatory cells. Over the past few years, evidence shows that both changes in stromal behaviour and the interaction between tumour cells and stromal cells are intimately linked to the processes of tumorigenesis, tumour invasion and metastasis (Liotta and Kohn, 2001; Bhowmick *et al*, 2004). Infact, it is now known that in addition to their production by epithelial tumour cells, MMP and/or TIMP gene expression may be induced in stromal fibroblasts and/or in vascular and inflammatory cells that infiltrate tumours around them (Visscher *et al*, 1994; Nielsen *et al*, 2001; Nakopoulou *et al*, 2002b; De Wever and Mareel, 2003). Likewise, a number of reports have indicated that the main source of MMPs in breast carcinoma is the stromal cell (Soini *et al*, 1994; Heppner *et al*, 1996; Brummer *et al*, 1999; Nakopoulou *et al*, 1999), and experimental studies have also demonstrated a mechanism by which breast cancer cells can rapidly use MMPs produced by adjacent normal fibroblasts to facilitate their invasion of the peritumoural tissue (Saad *et al*, 2002). In any case, in the present study it especially relevant to the finding of a positive and significant relationship between the expression of components of the matrix-degrading protease system by stromal cells of the tumours, such as fibroblasts or inflammatory cells, and several parameters indicators of tumoural aggressiveness, such as large size, lymph node involvement, high Nottingham Prognostic Index, infiltrating edge, desmoplastic reaction or peritumoural inflammation. In addition, we identify a subset of breast tumours having a phenotype of inflammatory mononuclear cells expressing MMPs and TIMPs, which was notably associated with a high relapse rate of distant metastasis. Therefore, this suggests that the tumoural stroma does not merely play a passive role in cancer progression. Rather, it may in fact actively participate in the process of cancer invasion.

Inflammatory cells can account for as much as 50% of the total tumour mass in invasive breast carcinomas. Mononuclear inflammatory cells infiltrate in breast carcinomas include a variable representation of macrophages, plasma cells, mast cells and B and T lymphocytes (Coussens and Werb, 2002; Lin and Pollard, 2004). Historically, tumour-infiltrating leukocytes have been considered to be manifestations of an intrinsic defence mechanism against developing tumours (Johnson *et al*, 1989; Lin and Pollard, 2004). However, our data are in accordance with the increasing evidence indicating that leukocytes infiltration can promote tumour phenotypes, such as angiogenesis, growth and invasion (Adams *et al*, 1987; Coussens and Werb, 2002; Daniel *et al*, 2005). This may be due to inflammatory cells probably influencing cancer promotion by secreting cytokines, growth factors, chemokines and proteases, which stimulate proliferation invasiveness of cancer cells. Nevertheless, the prognostic significance of the lymphoid infiltrate at the tumour site remains controversial, perhaps because the evaluation criteria for tumour infiltrates are not sufficiently standardised to yield reliable and reproducible results in different institutions. Therefore, our results may contribute to characterise a phenotype of infiltrating mononuclear inflammatory cells associated with a poorer prognosis in breast cancer. These mononuclear inflammatory cells were characterised by the expression of MMP-7, -9, -11, -13, -14, TIMP-1 and -2.

Matrix metalloproteinase-7 (matrilysin 1) is a stromelysin, which degrades type IV collagen, fibronectin and laminin. It was showed that MMP-7 is aberrantly expressed in human breast tumours, and that elimination of MMP-7 is associated with low invasiveness and slow tumour growth (Jiang *et al*, 2005). Matrix metalloproteinase-9 (gelatinase B) is related to tumour invasion and metastasis by their special capacity to degrade the type IV collagen found in basement membranes (Jones and Walker, 1997), and to induce angiogenesis (Egeblad and Werb, 2002). High MMP-9 expression correlates significantly with tumoural aggressiveness and poor prognosis (Chantrain *et al*, 2004; Li *et al*, 2004; Pellikainen *et al*, 2004). Similarly to other studies, our data showed that MMP-11 (Stromalysin-3) was preferentially expressed by peritumoural stromal cells (Basset *et al*, 1990, 1997) and that high levels of MMP-11 were associated with tumour progression and poor prognosis (Muller *et al*, 1993; Anderson *et al*, 1995; Chenard *et al*, 1996; Ahmad *et al*, 1998; Tetu *et al*, 1998; Lochter *et al*, 1999; Nakopoulou *et al*, 2002b). Matrix metalloproteinase-13 (collagenase-3) has been found to have an exceptionally wide substrate specificity when compared with other MMPs (Freije *et al*, 1994; Knauper *et al*, 1997). Moreover, it is thought to play a central role in the MMP activation cascade, both activating and being activated by several other MMPs (MMP-14, -2 or -3). Nielsen *et al* (2001) have reported that MMP-13 expression by myofibroblasts was often associated with microinvasive events, and they have proposed that this MMP may play an essential role during the transition of ductal carcinoma *in situ* lesions to invasive ductal carcinoma of the breast. Matrix metalloproteinase-14 (membrane type 1 MMP or MT1-MMP) is a key metalloprotease involved in the degradation of extracellular matrix, activates pro-MMP-13 (Knauper *et al*, 1996) and pro-MMP-2 (Strongin *et al*, 1995) on the cell surface, and plays crucial roles in molecular carcinogenesis, tumour cell growth, invasion and angiogenesis. On the other hand, the positive relationship between TIMP expression by inflammatory mononuclear cells and cancer progression could look paradoxical, because both TIMP-1 and -2 are well-known inhibitors of MMP activity. If TIMPs inhibit MMPs *in vivo*, it should be expected that high levels of inhibitors would prevent tumour progression and thus relate to good outcome in patients with cancer. However, there is an increasing body of evidence suggesting that TIMPs are multifunctional proteins that, in addition to its MMP-inhibitory effect, also promote the proliferation of some cell types, and their antiapoptotic effects may favour tumour expansion during the onset and early growth of the primary tumour (Guedez *et al*, 1998, 2001; Baker *et al*, 1999; Jiang *et al*, 2002).

The unresolved question is, if the mononuclear inflammatory cells merely respond to signals from the carcinoma cells or respond in self-addressed envelope on tumour progression. Even so, in the present study, we identify a phenotype of mononuclear inflammatory cells infiltrating tumours, characterised by the expression of a specific panel of MMPs and TIMPs, which is associated with the development of distant metastasis. Therefore, this finding is of great clinical interest and it also suggests that these host inflammatory cells could be a possible target for inhibition of tumour progression and metastasis.

## Figures and Tables

**Table 1:** Expression of MMPs and TIMPs in two cluster groups of breast carcinomas with distinct MMP/TIMP molecular profile in the stromal mononuclear inflammatory cells

	**Group 1 *n*=89**	**Group 2 *n*=42**	***P*-value**
MMP-1	53 (61.6)	32 (76.2)	NS
MMP-2	1 (1.1)	1 (2.4)	NS
MMP-7	33 (38.8)	30 (71.4)	0.001
MMP-9	0	14 (33.3)	0.0001
MMP-11	4 (4.6)	36 (85.7)	0.0001
MMP-13	21 (23.6)	23 (54.8)	0.001
MMP-14	29 (32.6)	38 (90.5)	0.0001
TIMP-1	9 (10.1	24 (57.1)	0.0001
TIMP-2	15 (16.9)	35 (83.3)	0.0001
TIMP-3	43 (48.3)	26 (61.9)	NS

Data are expressed as number of cases (%).

Note: samples on tissue sections were either insufficient or lost for analysis in three cases for MMP1, two cases for MMP-2, four cases for MMP-7, one case for MMP-9, two cases for MMP-11, one case for TIMP-1 and one case for TIMP-2. The values shown corresponding to the total number of cases were analysed for each protein.

**Table 2:** Number and percentage of positive cases of tumours for the different MMPs and TIMPs expressions, in tumour cells or fibroblastic cells, in the different cluster groups

	**Group 1 *n*=89**	**Group 2 *n*=42**	***P*-value**
*MMP-7*			
TC	72 (84.7)	32 (92.9)	NS
FC	57 (67.1)	33 (78.6)	NS
MIC	33 (38.8)	30 (71.4)	0.001
			
*MMP-9*			
TC	57 (64.8)	38 (90.5)	0.004
FC	6 (6.8)	14 (33.3)	<0.001
MIC	0	14 (33.3)	<0.001

*MMP-11*			
TC	73 (83.9)	41 (97.6)	0.04
FC	48 (55.2)	40 (95.2)	<0.001
MIC	4 (4.6)	36 (85.7)	<0.001

*MMP-13*			
TC	65 (73)	32 (76.2)	NS
FC	36 (40.4)	28 (66.7)	0.009
MIC	21 (23.6)	23 (54.8)	0.001

*MMP-14*			
TC	77 (86.5)	41 (97.6)	NS
FC	67 (75.3)	39 (92.9)	0.03
MIC	29 (32.6)	38 (90.5)	<0.001

*TIMP-1*			
TC	82 (92.1)	41 (97.6)	NS
FC	41 (46.1)	23 (54.8)	NS
MIC	9 (10.1)	24 (57.1)	<0.001
			
*TIMP-2*			
TC	69 (77.5)	41 (97.6)	0.008
FC	21 (23.6)	34 (81)	<0.001
MIC	15 (16.9)	35 (83.3)	<0.001

*TIMP-3*			
TC	75 (84.3)	38 (90.5)	NS
FC	46 (51.7)	34 (81)	0.003
MIC	43 (48.3)	26 (61.9)	NS

F=fibroblasts; MIC=mononuclear inflammatory cells; MMP=matrix metalloproteinase; TC=tumoural cells; TIMP=tissular inhibitors of metalloproteinase.

Data are expressed as number of cases (%).

Note: samples on tissue sections were either insufficient or lost for analysis in four cases for MMP-7, one case for MMP-9, two cases for MMP-11, one case for TIMP-1 and one case for TIMP-2. The values shown corresponding to the total number of cases were analysed for each protein.
